# Performance Evaluation of Precise Point Positioning for BeiDou-3 B1c/B2a Signals in the Global Range

**DOI:** 10.3390/s21175780

**Published:** 2021-08-27

**Authors:** Ershen Wang, Tao Yang, Zhi Wang, Yize Zhang, Jing Guo, Wansen Shu, Pingping Qu

**Affiliations:** 1 School of Electronic and Information Engineering, Shenyang Aerospace University, Shenyang 110136, China; wanges_2016@126.com (E.W.); ytao2021@126.com (T.Y.); shu_ws1202@126.com (W.S.); qupingping_79@163.com (P.Q.); 2 Zhejiang Key Laboratory of General Aviation Operation Technology, Jiande 311612, China; wangzhi@camic.cn; 3 Civil Aviation Management Institute of China, Beijing 100102, China; 4 Shanghai Astronomical Observatory, Chinese Academy of Sciences, Shanghai 200030, China; 5 China Academy of Civil Aviation Science and Technology, Beijing 100028, China; guoj@mail.castc.org.cn

**Keywords:** BDS-3, B1C/B2a signal, precise point positioning, performance evaluation, positioning accuracy

## Abstract

With the construction and development of the BeiDou navigation satellite system (BDS), the precise point positioning (PPP) performance of the BDS is worthy of research. In this study, observational data from 17 stations around the world across 20 days are used to comprehensively evaluate the PPP performance of BDS B1c/B2a signals. For greater understanding, the results are also compared with the Global Positioning System (GPS) and BDS PPP performance of different signals and system combinations. The evaluation found root mean square (RMS) values of the static PPP in the north (N), east (E), and upward (U) components, based on the B1c/B2a frequency of BDS-3, to be 6.9 mm, 4.7 mm, and 26.6 mm, respectively. Similar to the static positioning, the RMS values of kinematic PPP in the three directions of N, E, and U are 2.6 cm, 6.0 cm, and 8.5 cm, respectively. Besides this, the static PPP of BDS-3 (B1cB2a) and BDS-2 + BDS-3 (B1IB3I) have obvious system bias. Compared with static PPP, kinematic PPP is more sensitive to the number of satellites, and the coordinate accuracy in three dimensions can be increased by 27% with the combination of GPS (L1L2) and BDS. Compared with BDS-2+BDS-3 (B1IB3I), the convergence time of BDS-3 (B1CB2a) performs better in both static and kinematic modes. The antenna model does not show a significant difference in terms of the effect of the convergence speed, though the number of satellites observed has a certain influence on the convergence time.

## 1. Introduction

The BeiDou Navigation Satellite System (BDS) is a global satellite navigation system independently developed and operated by China. On 31 July 2020, The BDS-3 global satellite navigation system was officially announced to provide a global service [1,2]. In addition to the traditional B1I and B3I signals, BDS-3 also transmits several new signals with advanced signal structures, namely, B1C, B2a, B2b, and B2a + b. In terms of signal design, B1C signals are compatible with GPS L1C and Galileo E1 signals, while B2a signals are compatible with GPS L5C and Galileo E5a signals. The new B2b signal is used for smooth transition and compatibility with BDS-2, which can achieve better performance and is competitive with other GNSS signals [3]. Compared with BDS-2, the BDS-3 constellation is composed of three Geostationary Earth Orbit (GEO) satellites, three Inclined Geosynchronous Satellite Orbit (IGSO) satellites, and twenty-four Medium Earth Orbit (MEO) satellites, and these satellites of BDS-3 are manufactured by the China Academy of Space Technology (CAST) and Shanghai Engineering Center for Microsatellites (SECM) [4].

Precision point positioning (PPP) aims to provide positioning accuracy from decimeters to centimeters through global navigation satellite systems. Researchers from Universities and Institutes in China and abroad have carried out in-depth research into BDS-2, such as studies into the signal characteristics of the code observations [5,6], pseudo-range variations [7,8], precision clock estimation (PCE) [9], positioning performance [10,11,12]. inter-satellite-type biases (ISTBs) [13,14], precise orbits and clocks [15,16], and so on.

In recent years, numerous research projects have been conducted on the BDS-3 precise clock estimation [17,18,19], time group delay (TGD)/differential code bias (DCB) [20,21], PPP-B2b signal evaluation [22,23,24], and satellite availability [25,26]. Whether from the composition of the signal or the signal propagation, positioning is fundamental. Although the full operation of BDS-3 began in July 2020, the PPP performance of BDS-3 on a global scale has rarely been analyzed. Jiao et al. [27] evaluated the PPP performance for the combination of BDS-3 and BDS-2. The RMS values of the static BDS-2/BDS-3 PPP were 10.7 cm, 19.5 cm, and 20.4 mm, respectively, in the geographic area of the selected station, which is the same level as GPS and GLONASS. The RMS values of the kinematic BDS-2/BDS-3 PPP were 5.88 cm, 7.43 cm, and 9.50 cm, respectively. The positioning accuracy, convergence time, and zenith tropospheric delay are also used to verify the performance of PPP, but only the stations in Asia and Europe are selected, though most are located in Asia. Su et al. [28] analyzed the performance of the combination of quad-frequency PPP models of BDS and Galileo, and the results show that quad-frequency PPP generates a significant improvement when compared with single-frequency. The pseudo-range noise of the new frequency of B1C and B2a is slightly larger than that of the B1I and B3I signals. Mainly, however, researchers use data concentrated on one station for analysis. Wang and Hong et al. [29,30] compared the performance of low-cost single-frequency precise point positioning (SFPPP), starting from the aspects of positioning accuracy and convergence speed, but only the single-frequency model was considered. Some scholars have studied the performance of PPP from the orbital clock products provided by IGS [31] and the combination of multi-frequency PPP and multi-system PPP [32,33], but the analysis focused on more the combination PPP of B1I and B3I frequencies, and focused less on the analysis of B1C and B2a frequencies PPP. Zhu et al. [34] simply analyzed the positioning performance of B1C and B2a from static PPP, but did not conduct a comprehensive analysis of PPP. The results showed that the horizontal positioning errors were about 1.38~4.42 cm, and the vertical positioning errors were about −1.31~4.34 cm in the mid-latitude area after convergence. Jin et al. [35] used BDS B1I, B3I, B1C, and B2a to evaluate the performance of the BeiDou PPP model of a single frequency, dual frequency, triple frequency, and quad frequency, with theoretical comparison of the models, positioning performances, precise time transfer, zenith tropospheric delay (ZTD), inter-frequency bias (IFB), and differential code bias (DCB), but the station was only analyzed on one day. Although the above-mentioned literature carried out some research into the PPP positioning performance of BDS-3, there are few comprehensive positioning performance evaluations of the B1c and B2a frequencies on a global scale. Evaluation of the new signals (B1C/B2a) can expand our understanding of the performance of BDS-3 (B1C/B2a) PPP in the global range, providing a basis for the subsequent combination of three-frequency or four-frequency positioning, thereby increasing the effect of global positioning.

In this paper, the B1C and B2a dual-frequency signals of BDS-3 are used to analyze the global PPP performance of BDS. Twenty days of observation data from 17 stations distributed around the world are used to analyze the positioning accuracy and convergence performance of kinematic and static PPP. The PPP performances of GPS, BDS-2 + 3 (B1IB3I), GPS + BDS-3 (B1cB2a), and GPS+BDS-2 + 3 (B1IB3I) are also compared.

## 2. Model and Parameter Estimation

### 2.1. Positioning Function Model

GNSS observations are affected by various errors related to hardware equipment, the propagation process, and observation environment. The observation models are given, regarding the mathematical relationship between observations, observation errors, and parameters [36,37,38]. At present, the widely used PPP models include ionosphere-free PPP and ionosphere-weighted PPP [29,39,40,41,42]. Each model has its own advantages. In this paper, a combination of ionospheric-free PPP models is used.

The basic observation equations of the BDS ionosphere-free carrier phase and pseudo-range can be expressed as

(1) $$\begin{array}{c} P_{r,IF}^{s,T}=\rho^{s,T}+c\cdot dt_{r}-c\cdot dt^{s,T}+T_{r}^{s,T}+B_{r,P_{IF}}^{T}-B_{P_{IF}}^{s,T}+Re_{r}^{s,T}+Er_{r}^{s,T}+Pco_{r,IF}^{s,T}\\ +Pcv_{r,IF}^{s,T}+Tid_{r}^{s,T}+\varepsilon_{P_{IF}}^{s,T} \end{array}$$

(2) $$\begin{array}{c} L_{r,IF}^{s,T}=\lambda_{IF}\Phi_{IF}^{s,T}=\rho^{s,T}+cdt_{r}-cdt^{s,T}+T_{r}^{s,T}+\lambda_{IF}(N_{IF}^{s,T}+B_{r,L_{IF}}^{T}-B_{L_{IF}}^{s,T}+W_{IF}^{s,T})+Re_{r}^{s,T}+Er_{r}^{s,T}\\ +Pco_{r,IF}^{s,T}+Pcv_{r,IF}^{s,T}+Tid_{r}^{s,T}+\varepsilon_{L_{IF}}^{s,T} \end{array}$$

where the subscript r and superscript s refer to the receiver and satellite, respectively; the subscript IF denotes ionosphere-free; the superscript T denotes the satellite system; P and L are the pseudo-range observations (m) and the phase observation (m), respectively; ρ is the geometric distance from the satellite to receiver (m); c is the speed of light in a vacuum (s); $dt_{r}$ and $dt^{s}$ are the receiver clock offset and satellite clock offset (m/s), respectively; T is the tropospheric delay on the signal propagation path (m); λ is the wavelength (m); Φ is the phase observations in cycles; N is the ambiguity of the carrier phase; $B_{r}$ and $B_{}^{s}$ are the pseudo-range hardware delays at the receiver and satellite sides (m), respectively; W is the phase wind-up error in cycles; Re is the relativistic effect caused by satellite track eccentricity (m); Er is the error caused by the Earth’s rotation; Pco and Pcv are the phase center offset (PCO) and phase center variation (PCV) of the receiver and satellite; Tid is the earth tide error; and ε are the unmodeled errors (multipath, noise).

(3) $$\rho =\sqrt{{\left(x_{r}-x^{s}\right)}^{2}+{\left(y_{r}-y^{s}\right)}^{2}+{\left(z_{r}-z^{s}\right)}^{2}}$$

where $\left[x_{r},y_{r},z_{r}\right]$ and $\left[x^{s},y^{s},z^{s}\right]$ are the coordinates of the receiver and satellite respectively. Then, linearize ρ at its approximate coordinate $\left[x_{0},y_{0},z_{0}\right]$, which can be written as

(4) $$\rho =\rho_{0}+\frac{x_{0}-x^{s}}{\rho_{0}}dx+\frac{y_{0}-y^{s}}{\rho_{0}}dy+\frac{z_{0}-z^{s}}{\rho_{0}}dz$$

where $\rho_{0}$ is the geometric distance from the satellite to the receiver’s approximate coordinates.

Observations are used to establish the PPP model, but the accuracy of the tropospheric model does not meet the requirements of PPP. Hence, the residual part of the zenith tropospheric wet delay is normally estimated together with station coordinates and carrier phase ambiguity. However, other error corrections, such as PCO, PCV, phase wind-up, relativistic effect, etc., are corrected by corresponding methods. The unknown parameters are the receiver coordinates and clock errors, zenith tropospheric wet delay, and phase ambiguity. Therefore, the observation equation of PPP is formed as:

(5) $$\left[\begin{array}{c} P_{1}-\rho_{1}-D_{P1}\\ L_{1}-\rho_{1}-D_{L1}\\ \vdots \\ P_{n}-\rho_{n}-D_{Pn}\\ L_{n}-\rho_{n}-D_{Ln} \end{array}\right]=\left[\begin{array}{cccccccc} \frac{x_{0}-x^{1}}{\rho_{0}} & \frac{y_{0}-y^{1}}{\rho_{0}} & \frac{z_{0}-z^{1}}{\rho_{0}} & 1 & M_{wet}^{1} & 0 & \cdots & 0\\ \frac{x_{0}-x^{1}}{\rho_{0}} & \frac{y_{0}-y^{1}}{\rho_{0}} & \frac{z_{0}-z^{1}}{\rho_{0}} & 1 & M_{wet}^{1} & 1 & \cdots & 0\\ \vdots & \vdots & \vdots & \vdots & \vdots & \vdots & \vdots & \vdots \\ \frac{x_{0}-x^{n}}{\rho_{0}} & \frac{y_{0}-y^{n}}{\rho_{0}} & \frac{z_{0}-z^{n}}{\rho_{0}} & 1 & M_{wet}^{n} & 0 & \cdots & 0\\ \frac{x_{0}-x^{n}}{\rho_{0}} & \frac{y_{0}-y^{n}}{\rho_{0}} & \frac{z_{0}-z^{n}}{\rho_{0}} & 1 & M_{wet}^{n} & 0 & \cdots & 1 \end{array}\right]\left[\begin{array}{c} dx\\ dy\\ dz\\ c.d\delta \\ dZTD_{\omega }\\ B_{1}\\ \vdots \\ B_{n} \end{array}\right]$$

where $M_{wet}$ is the tropospheric wet delay mapping function; $dZTD_{\omega }$ is the zenith tropospheric wet delay; B is the ambiguity parameter. Formula (5) is abbreviated as y=Gx.

### 2.2. Kalman Filtering

Kalman filtering is the most commonly used algorithm in GNSS positioning. Kalman filtering is based on the state estimation of the previous epoch combined with observations of the current epoch to recursive new state parameters with the state transition matrix. It is mainly divided into two steps: state prediction and parameter update. The prediction model can be expressed as

(6) $$\left\{\begin{array}{c} {\hat{x}}^{-}(k)=\Phi (k-1){\hat{x}}^{-}(k-1)\\ P_{\hat{x}(k)}^{-}=\Phi (k-1)P_{\hat{x}(k-1)}^{-}\Phi^{T}(k-1)+Q(k-1) \end{array}\right.$$

where k is the epoch; x is the estimated parameters; Φ is the state transition matrix from the previous epoch to the current epoch; Q is the process noise matrix; P is the covariance matrix of the parameters; $\hat{*}$ is the estimated value; ${*}^{-}$ is a priori value.

In Kalman filtering, the setting of kinematic and static modes is different. Differences are mainly manifested in the setting of state transition matrix Φ and process noise matrix Q [43,44].

For static PPP, the coordinate parameters are unchanged, the clock error parameter is a random noise model, and the tropospheric parameters are generally a random walk model. When there is no cycle slip, the ambiguity parameter remains unchanged, so the included coordinates and receiver clock error and the state transition matrix Φ and process noise matrix Q of the tropospheric wet delay and ambiguity parameters can be written as:

(7) $$\Phi =\left[\begin{array}{cccccc} 1 & & & & & \\ & 1 & & & & \\ & & 1 & & & \\ & & & 0 & & \\ & & & & 1 & \\ & & & & & 1 \end{array}\right],Q=\left[\begin{array}{cccccc} 0 & & & & & \\ & 0 & & & & \\ & & 0 & & & \\ & & & \sigma_{\delta t}^{2} & & \\ & & & & \sigma_{trop}^{2} & \\ & & & & & 0 \end{array}\right]$$

where $\sigma_{\delta t}^{}=1\;\mathrm{ms}$ and $\sigma_{trop}^{}=20\;\mathrm{mm}/\sqrt{\mathrm{hour}}$; when the phase cycle slips, the coefficient in the state transition matrix is 0.

For kinematic PPP, if its moving speed is unknown and the coordinate parameters are random noise models, its state transition matrix Φ and process noise matrix Q can be defined as:

(8) $$\Phi =\left[\begin{array}{cccccc} 0 & & & & & \\ & 0 & & & & \\ & & 0 & & & \\ & & & 0 & & \\ & & & & 1 & \\ & & & & & 1 \end{array}\right],Q=\left[\begin{array}{cccccc} \sigma_{dx}^{2} & & & & & \\ & \sigma_{dy}^{2} & & & & \\ & & \sigma_{dz}^{2} & & & \\ & & & \sigma_{\delta t}^{2} & & \\ & & & & \sigma_{trop}^{2} & \\ & & & & & 0 \end{array}\right]$$

where $\left[\sigma_{dx},\sigma_{dy},\sigma_{dz}\right]$ is the coordinate noise, and the kinematic PPP is set to 100 m.

The parameter update step can be expressed as

(9) $$\left\{\begin{array}{c} P_{\hat{X}(k)}=\left[I\;-\;K(k)G(k)\right]P_{\hat{X}(k)}^{-}\\ \hat{x}(k)={\hat{x}}^{-}(k)+\;K(k)\left[y(k)\;-\;G(k){\hat{x}}^{-}(k)\right] \end{array}\right.$$

where y is the observation minus correction; G is the observation design matrix; K(k) the Kalman gain, which can be expressed as

(10) $$K(k)=P_{\hat{X}(k)}^{-}G^{T}(k){\left[G(k)P_{\hat{X}(k)}^{-}G^{T}(k)+R(k)\right]}^{-1}$$

## 3. Experimental Evaluation

### 3.1. Data source and Configuration Analysis

**Figure 1:**
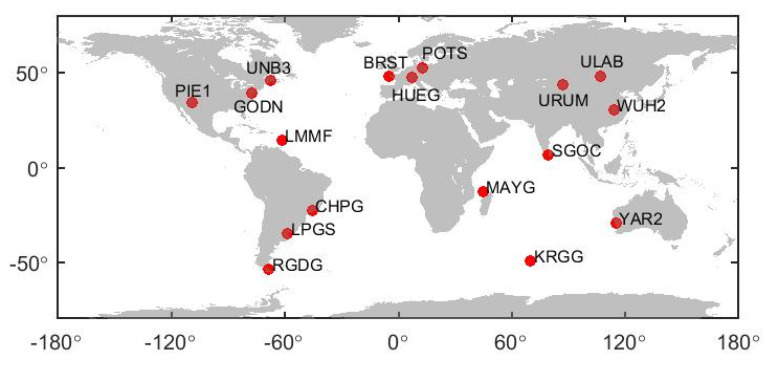
Monitoring station distribution.

To verify the performance of BDS PPP, observation data of 17 global stations from 1 December 2020 to 20 December 2020 were used. BDS B1CB2a, B1IB3I, and GPS signals can be received by the test stations. Taking a day at one station as a sample, there are in total 340 data samples. The observation data interval is 30 s. Since BDS-3 was built to serve the world, to ensure the objectivity of the evaluation, the selection of the stations around the world should be as uniform as possible. The detailed distribution of the stations is shown in Figure 1.

The precise ephemeris and orbit provided by the IGS analysis centers include final, rapid, ultra-rapid, and real-time (RTS) products. The accuracy of the products provided by each analysis center is slightly different [45]. The precise orbit and clock products used in this research are the GBM products provided by GFZ (Geo Forschungs Zentrum, Potsdam, Germany). The coordinate reference of the stations is the SNX file provided by IGS. It should be noted that the PCO and PCV corrections of some antennas of the receiver only provide corrections for GPS and GLONASS [27]. Therefore, the PCO and PCV corrections of GPS are used to correct for BDS.

The GNSS multi-function precision positioning software Net_Diff is used for PPP in this research (https://github.com/YizeZhang/Net_Diff) (accessed on 20 August 2021). The specific settings for PPP are shown in Table 1.

### 3.2. Experimental Process

In this study, the abnormal value of a single sample has been excluded, which refers to a single sample with a small number of satellites participating in the PPP calculation. The excluded samples for static PPP and kinematic PPP are shown in Table 2, where G, C12, and C13, respectively, represent the positioning modes of GPS(L1L2), BDS (B1cB2a), and BDS (B1IB3I). The number (DOY) + station name represents the day of year and the station. For example, 337PIE1 represents the sample of PIE1 on the 337th day in 2020, and “all” represents all days from 336–355.

**Figure 2:**
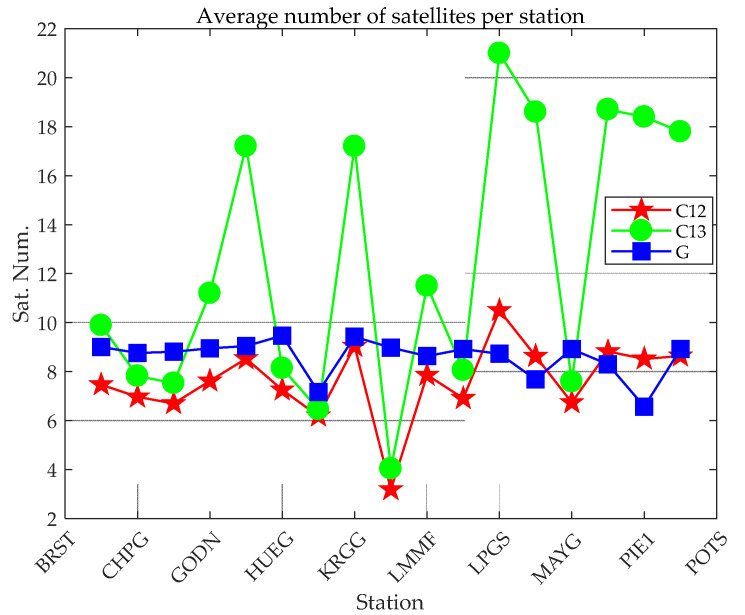
Average number of satellites participating in PPP at 17 stations.

For the same observation data, regardless of whether static PPP or kinematic PPP, the number of satellites involved in positioning is the same. This article counts the average number of satellites participating in BDS (B1CB2a), BDS (B1IB31), and GPS (L1L2) PPP for 17 stations, as shown in Figure 2. The PPP availability of this station can be better reflected by the average number of satellites. The stations with a small average number of satellites shown in Figure 2 can basically correspond to the stations excluded in Table 2.

**Figure 3:**
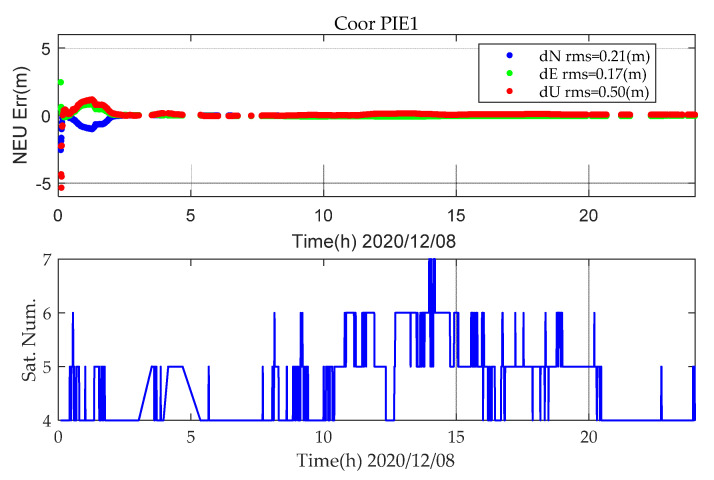
BDS (B1IB3I) static PPP positioning error and number of satellite (DOY343, 2020).

For the overall performance of static PPP, due to the limited number of satellites, there are large errors, which have a great impact on positioning statistics and lack statistical objectivity. Figure 3 shows the static PPP error at station PIE1 for BDS-2+3 (B1IB3I), and the number of satellites involved in positioning on DOY 343 in 2020. In Figure 3, there are less than five satellites participating in static PPP for a long time, and the position information cannot be calculated in static PPP.

**Figure 4:**
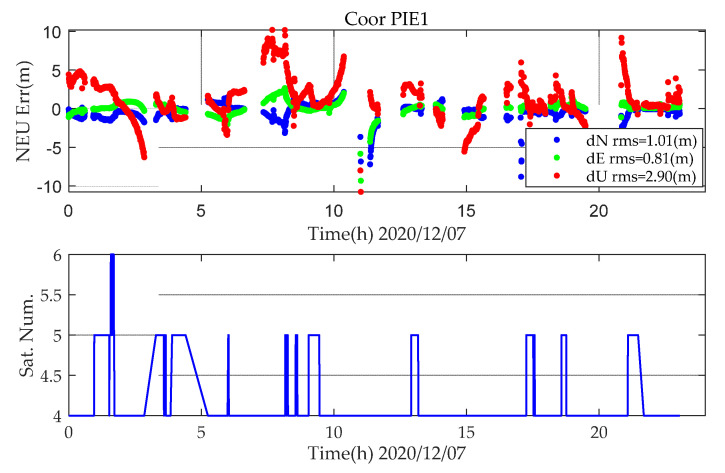
BDS (B1CB2a) kinematic PPP positioning error and number of satellite (DOY342,2020).

For kinematic PPP, the number of satellites has a very large impact on positioning. When the number of satellites is less than five, abnormal jumps occur, as shown in Figure 4.

Therefore, in the subsequent data analysis, the above cases are excluded.

### 3.3. Static PPP

**Figure 5:**
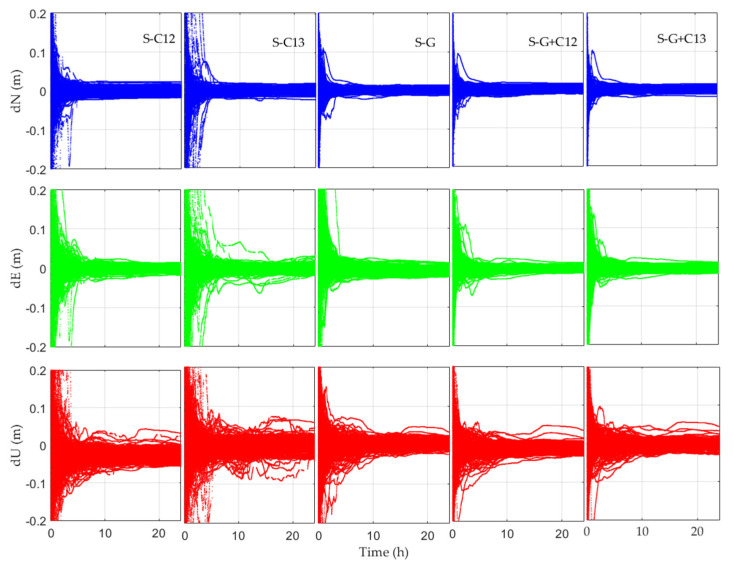
Static PPP error series for 20 days at 17 stations utilizing GBM’s precise orbit/clock corrections (the result after the abnormal data are raised).

The precise orbit and clock offset provided by GBM are used to perform static PPP of BDS (B1CB2a). Figure 5 demonstrates a static PPP error series for 20 days at 17 stations utilizing GBM’s precise orbit/clock corrections. All results for the 20 days at 17 stations are plotted in the same figure. The premise is to eliminate the abnormal values in Table 2, where S represents the results for static PPP. It can be seen that all PPP solutions can reach convergence but with significantly different convergence times. In the meantime, the positioning errors in the N and E directions are obviously smaller than those of the U component.

**Figure 6:**
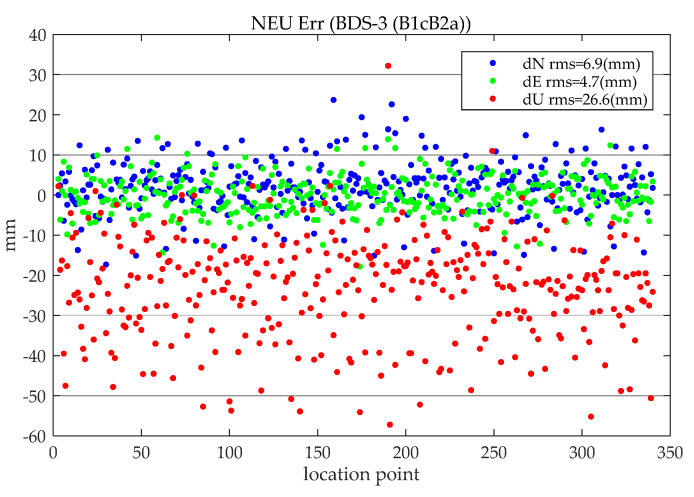
Static BDS-3 (B1cB2a) PPP positioning error.

When performing static PPP, the last epoch of a one-day solution is taken as the result of the static positioning of that day (mark with location point; no explanation is given for the subsequent static PPP position error statistics, and the same processing method is used). Figure 6 shows the distribution of the north (N), east (E), and upward (U) positioning errors of all stations on all days (BDS(B1cB2a)). It can be seen that the accuracies of BDS (B1cB2a) static PPP in the three directions of N, E, and U are 6.9 mm, 4.7 mm, and 26.6 mm, respectively. Compared with the horizontal error, the distribution of vertical error is more scattered.

To further compare and analyze the static PPP performance of BDS-3 (B1cB2a), GPS (L1L2), BDS-2+3 (B1IB3I), GPS + BDS-3 (B1cB2a), and GPS + BDS-2+3 (B1IB3I), static PPP are also processed. To facilitate the analysis of the gap between the frequency and system combination of BDS and GPS, the RMS values of BDS-3 (B1cB2a), BDS-2+3 (B1IB3I), GPS, GPS + BDS-3 (B1cB2a), and GPS + BDS-2+3 (B1IB3I) static PPP error in the north, east, upward, and horizontal directions, and in three-dimensions (3D), are calculated.

**Figure 7:**
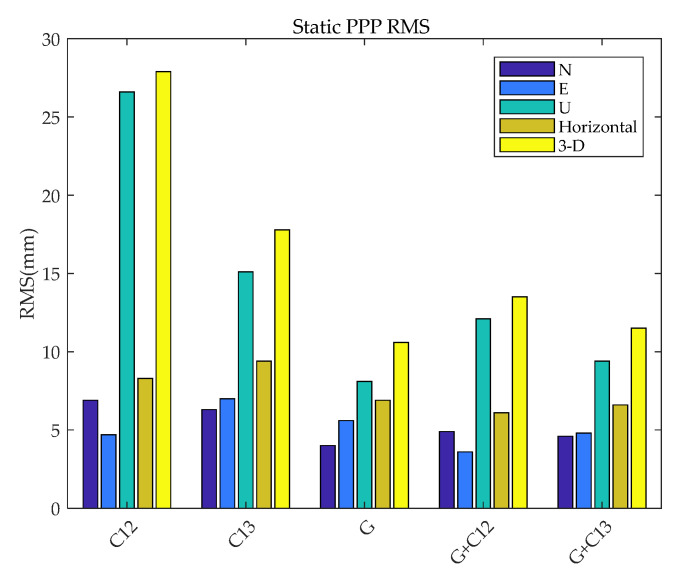
Static PPP positioning accuracy histogram.

The results are shown in Figure 7. The average values of the N, E, and U positioning errors for all the stations on all days are calculated and expressed by AVG, while the RMS of the positioning accuracy for static PPP is indicated by RMS. The results are shown in Table 3.

It can be seen from Table 3 and Figure 7 that the upward error of BDS-3 (B1cB2a) is larger than that of GPS and BDS-2+3 (B1IB3I). Compared with BDS (B1IB3I) and BDS-3 (B1cB2a), the horizontal error of GPS is 16.8% and 26.5% better, and the vertical component is 46.3% and 69.5% better, respectively. On the one hand, the precise orbit determination of BDS satellite PCO on-orbit estimation, precise attitude model, solar radiation pressure model, and other aspects of precise orbit determination strategies are not perfect yet, and using the correction values of GPS to correct the PCV and PCO of BDS will lead to significantly lower accuracy than that of GPS, especially in the vertical component. On the other hand, the number of satellites with B1C and B2a signals involved in static PPP is less than that of GPS and BDS-2+3 (B1IB3I). BDS-3 (B1cB2a) is significantly different from BDS-2+3 (B1IB3I) in terms of mean error (M-RMS). The mean error of BDS-2+3 (B1IB3I) is within 2mm and there is no large system error, while BDS-2+3 (B1IB3I) reached -23.2mm in the vertical direction, indicating a difference between PCO and PCV at different frequencies of BDS. Compared with the BDS-only system, the positioning accuracy of combined GPS and BDS is significantly improved. However, due to the influence of the BDS PCO correction issue, the performance of the vertical error is not ideal, and even slightly lower than the accuracy of GPS-only static PPP. Compared with BDS-3 (B1cB2a), GPS+BDS-3 (B1cB2a) increased by 27% and 52% in the horizonal and vertical directions, respectively.

### 3.4. Kinematic PPP

**Figure 8:**
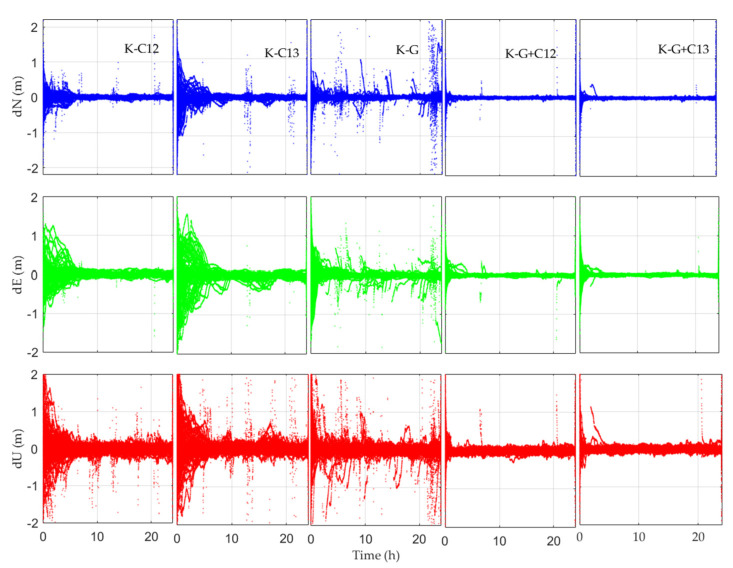
Kinematic PPP error series for 20 days at 17 stations utilizing GBM’s precise orbit/clock corrections (the result after the abnormal data are raised).

The kinematic solutions of all stations are shown in Figure 8, where K represents the results for kinematic PPP. Unlike the static solutions in Figure 5, the Y-axis in Figure 8 is expanded to ±2 m for a clearer view. Similar to static solutions, the kinematic positioning errors in the vertical component are larger than those of the horizontal component. However, the kinematic positioning errors are obviously larger when compared with the static solutions.

**Figure 9:**
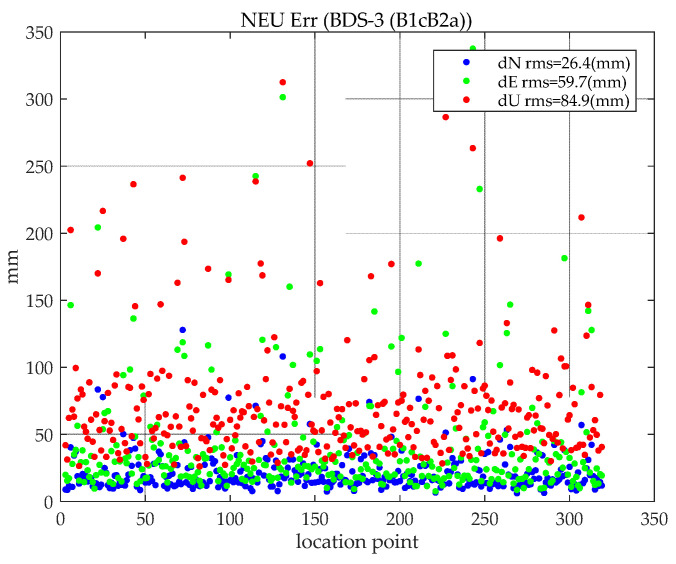
Kinematic BDS-3 (B1cB2a) PPP positioning RMS.

Similar to the evaluation method of static PPP, the positioning error within one hour after positioning is taken as the result of convergence, and the RMS of the PPP error after convergence is counted. The kinematic positioning results of BDS-3 (B1cB2a) are shown in Figure 9.

It can be seen from Figure 9 that the RMS values of the BDS-3 (B1cB2a) kinematic PPP results in the three directions of N, E, and U are 2.6, 6.0, and 8.5 cm, respectively. When the horizontal error is converged, the positioning accuracy reaches 6.5 cm, which proves that kinematic PPP can reach centimeter-level positioning accuracy.

**Figure 10:**
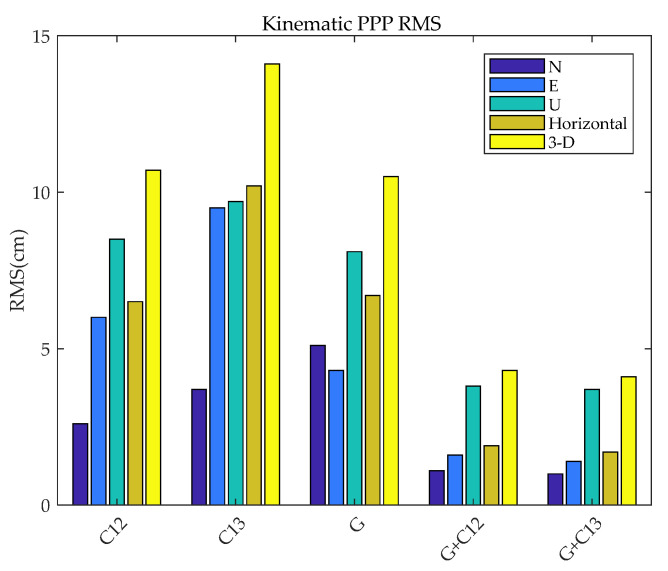
Kinematic PPP positioning accuracy histogram.

To better compare the results of BDS-3 (B1cB2a) kinematic PPP, the RMS of kinematic PPP are calculated for BDS-3 (B1cB2a), GPS, BDS-2+3 (B1IB3I), GPS + BDS-3 (B1cB2a), and GPS + BDS-2+3 (B1IB3I) in the N, E, U, and horizontal directions, and in 3D, as shown in Figure 10.

Figure 10 shows that compared with BDS-2+3 (B1IB3I), kinematic PPP of BDS-3 (B1cB2a) reduces the positioning errors of N, E, U, and 3-D error by 29%, 36%, 12%, and 24%, respectively. It can be seen that the observation value of B1cB2a is more accurate. The kinematic PPP accuracy of BDS-3 (B1cB2a) is slightly lower than that of GPS. The reason is that the satellite orbits and clock products provided by GBM do not include all BDS satellites, resulting in a relatively small number of satellites that can be used in positioning. On the other hand, the accuracy of the BDS orbit and clock is worse than that of GPS. Different from the impact of static PPP, the number of satellites has a larger impact on kinematic PPP. For the dual-system combination, due to the increase in the number of satellites, the 3-D error is increased by 59% and 70%, respectively, relative to BDS-3 (B1cB2a) and BDS-2+3 (B1IB3I). The effect of multi-GNSS on improving the positioning accuracy is more obvious in kinematic positioning. Considering that the receiver coordinates are estimated as white noise, the receiver coordinate parameters cannot be constrained by the explicit kinematics model as in the static processing mode. Therefore, increasing redundant observations can increase the reliability of kinematic solutions [9].

### 3.5. Convergency

Compared with RTK, PPP requires a longer convergence time to achieve centimeter-level positioning accuracy. In this section, factors of kinematic and static PPP convergence time are discussed from the previous hourly convergence performance and receiver antenna type.

**Figure 11:**
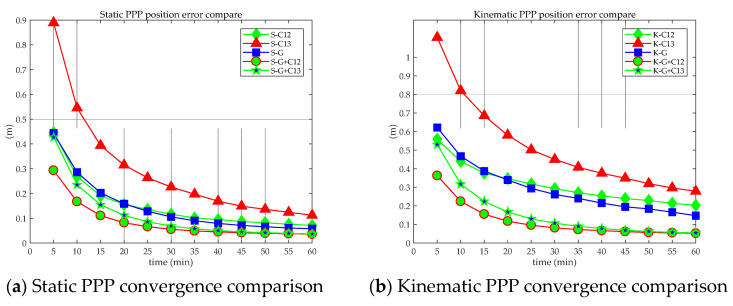
Positioning convergence in the first hour of static (**a**) and kinematic (**b**) PPP.

From the positioning results of the first hour of static and kinematic BDS-3 (B1cB2a), BDS-2+3 (B1IB3I), GPS, GPS + BDS-3 (B1cB2a), and GPS + BDS-2+3 (B1IB3I) PPP, the average 3D positioning errors of all stations on all days were calculated, which are shown in Figure 11, where S means static, K means kinematic, B means BDS, G means GPS, 12 means B1cB2a, and 13 means B1IB3I.

**Figure 12:**
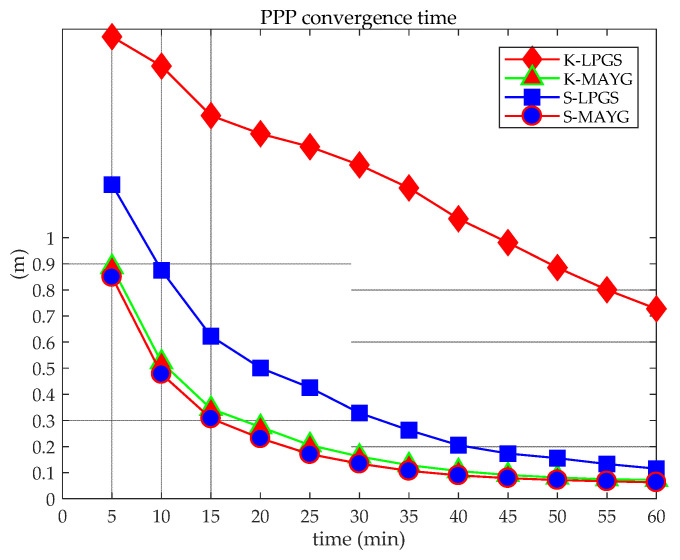
Positioning convergence in the first hour of LPGS and MAYG stations perform PPP.

Figure 11 shows that the convergence efficiency of the static PPP of a single system is significantly higher than that of a kinematic one. In addition, the differences between different systems are also obvious. Among them, whether it is static or kinematic PPP, the convergence effect of BDS (B1IB3I) is worse those that of GPS and BDS (B1CB2a), mainly due to the number of satellites observed by individual stations is small, which leads to poor overall convergence, as shown in Figure 2 and Figure 12. When the number of observation satellites is the same, because the observation noise of BDS (B1IB3I) is relatively large, the error of BDS (B1IB3I) PPP is greater than that of BDS (B1CB2a) PPP. Compared with the single system, the dual system has significantly improved the convergence efficiency. Static PPP of BDS (BICB2a) and GPS can converge to 0.1 m within 40 min, and the combined static PPP of BDS-3 (B1cB2a) and GPS can converge to 0.1 m within 20 min. For kinematic PPP, the single-constellation PPP did not converge to 0.1 m within 60 min. The kinematic PPP of BDS-3 (B1cB2a) + GPS and BDS-2+3 (B1IB3I) + GPS can converge to 0.1 m within 30 min and 35 min, respectively. Similar to the positioning accuracy, the number of satellites is an important factor in the convergence time of PPP. Under the condition of a large number of satellites (more than four satellites), GPS has the best convergence efficiency. For the positioning accuracy and convergence time, the performances of GPS + BDS (B1cB2a) or GPS + BDS (B1IB3I) PPP are superior to the single-constellation PPP.

To further analyze the convergence of BDS-3 (B1cB2a), the receiver types were classified and counted, and the positioning errors of each station at different epochs were observed. Table 4 shows the receiver model and antenna model at each station.

**Figure 13:**
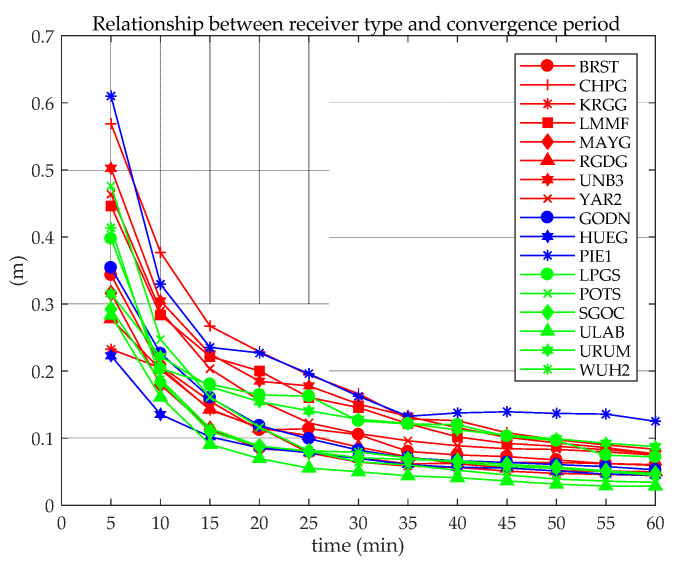
Positioning error in the previous hour for different receiver types (red: TRIMBLE ALLOY; blue: JAVAD TRE_3 DELTA; green: JAVAD TRE_3).

The 20-day data are used for static PPP, and the positioning error of each station during the first hour is analyzed according to the receiver type, as shown in Figure 13.

**Figure 14:**
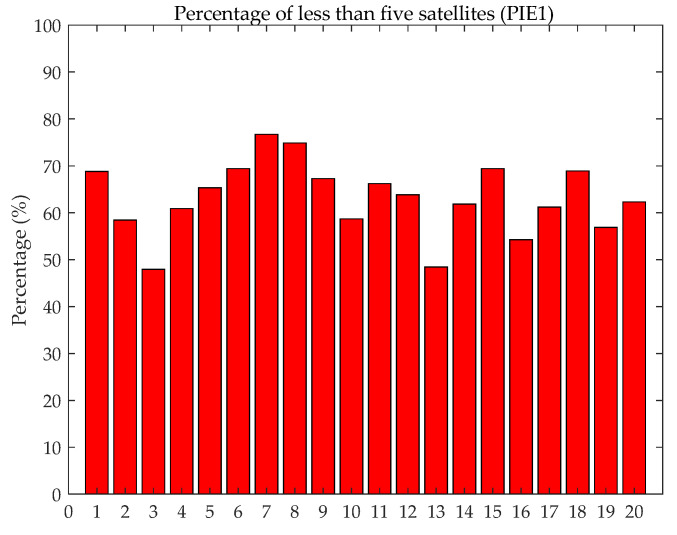
Fewer than five satellites participating in static PPP at PIE1 station accounted for the total epoch (20 days).

Figure 13 shows that there is no obvious difference between the receiver type and the convergence time. The main reason is the technological advancement of various manufacturers, and the antenna of the high-end receiver has little effect on BDS (B1CB2a) PPP. The PPP convergence speed is closely related to the number of satellites. Figure 14 shows the percentage with less than five satellites participating in static PPP at the PIE1 station in a day (20 day in total). Compared with other stations, the static PPP of PIE1 station has a poorer convergence performance, as shown in Figure 13. The percentage of PIE1 stations with less than five satellites participating in the 20-day PPP solutions is more than 40%, and the static PPP does not converge to less than 0.1 m within 60 min.

## 4. Conclusions

In this study, through the GNSS multifunctional precision positioning software Net_Diff, 20-day data for 17 stations in global were assessed to analyze the BDS-3 (B1cB2a) static and kinematic PPP performance. The BDS-2+3 (B1IB3I), GPS, GPS+BDS-3 (B1cB2a), and GPS + BDS-2+3 (B1IB3I) positioning error, static and kinematic convergence, and convergence efficiency between different receiver models were compared. The conclusions are summarized as follows.

(1) In terms of PPP error, the results show that the RMS accuracies of BDS (B1cB2a) static PPP are 6.9 mm, 5.2 mm, and 27.2 mm, and of kinematic PPP are 2.6 cm, 6.0 cm, and 8.5 cm in the North, East and Upward directions, respectively. Whether it is static PPP or kinematic PPP, the positioning accuracy of BDS (B1CB2a) and BDS (B1IB3I) PPP are worse than that of GPS. The main reason is that the accuracy of BeiDou’s precise orbit and clock difference provided by GBM is worse than that of GPS, and the BeiDou PCO correction model uses GPS correction parameters. In static PPP, BDS (B1cB2a) and BDS (B1IB3I) have obvious inter-system bias, which is mainly reflected in the upward. Kinematic PPP has a greater impact on the number of satellites.
(2) In terms of Convergency, for static PPP or kinematic PPP, BDS + GPS has better convergence than GPS only or BDS only. The main reason is the increase in the number of satellites involved in positioning. For BDS (BICB2a) PPP, there is no obvious difference in convergence between different antenna models (high-end receiver), though the number of satellites observed has a great impact on convergence. Because the observation noise of BDS (B1IB3I) is larger than that of BDS (B1CB2a), when the number of satellites is the same, the convergence of BDS (B1IB3I) is worse than that of BDS (B1CB2a). When the number of satellites participating in static PPP is less than five satellites and there are too many epochs of the total epochs in a day, the static PPP convergence does not reach 0.1 m within 60 min.

To sum up, BDS (B1CB2a) PPP can meet the PPP needs of BeiDou users in the global range, but compared with GPS, the elevation direction BDS (B1CB2a) PPP performs poorly. With the advancement of algorithms, these gaps will gradually narrow. BDS (B1CB2a) is equivalent to GPS in terms of convergence.

## Figures and Tables

**Table 1:** Precise point positioning configuration parameters.

	Parameters	Models
**Observation**	Observations	GPS: L1 & L2, BDS: B1I & B3I, BDS: B1c & B2a
	Elevation cutoff	10°
	Observation weighting	Weight determination of the elevation angle
	Standard deviation of observation noise	Phase 1 cm, pseudo range 1 m
	Sampling rate	30 s
**Error** **correction**	Satellite orbit	GBM precise orbit
	Satellite clock	GBM precise clock
	Phase wind-up	Model corrected [46]
	Phase center offset	igs14.atx
	Earth deformation	IERS conventions
	Ionospheric correction	Ionosphere-free combination
	Tropospheric correction	GPT2_5W + SAAS + VMF1
**Parameter** **estimation**	Ambiguity	Piecewise constant, float solution
	Troposphere	Random walk $\sigma_{trop}^{}=2\;\mathrm{cm}/\sqrt{\mathrm{hour}}$
	Station coordinates	Static: constant, kinematic: random walk (Set to 100 m)
	Clock correction	White noise

**Table 2:** Statistics of excluded sample values.

Static PPP		Kinematic PPP		
C12	C13	C12	C13	G
337PIE1 341PIE1	337PIE1 340PIE1 341PIE1 343PIE1 352PIE1 353PIE1	allPIE1 337LPGS 340LMMF 341UNB3	allPIE1 338LPGS 340CHPG 341UNB3 344CHPG 347CHPG 355GODN	336ULAB
				339SGOC
				341WUH2
				342ULAB
				342URUM
				344URUM
				345ULAB
				345WUH2
				346WUH2
				347URUM
				349WUH2
				351WUH2
				355URUM

**Table 3:** Static PPP positioning accuracy and average RMS value statistics.

		S-C12	S-C13	S-G	S-G+C12	S-G+C13
**RMS** **(mm)**	North	6.9	6.3	4.0	4.9	4.6
	East	4.7	7.0	5.6	3.6	4.8
	Upward	26.6	15.1	8.1	12.1	9.4
	Horizontal	8.3	9.4	6.9	6.1	6.6
	3-D	27.9	17.8	10.6	13.5	11.5
**AVG** **(mm)**	North	2.1	1.2	0.9	1.5	1.0
	East	−0.2	−0.7	−2.2	−0.9	−1.2
	Upward	−23.2	2.0	2.5	−8.6	0.7

**Table 4:** Monitoring station receiver type and antenna type.

Station Name	Receiver Model	Antenna Model
BRST	TRIMBLE ALLOY	TRM57971.00-NONE
CHPG	TRIMBLE ALLOY	TRM59800.00-NONE
GODN	JAVAD TRE_3 DELTA	TPSCR.G3-SCIS
HUEG	JAVAD TRE_3 DELTA	LEIAR25.R4-LEIT
KRGG	TRIMBLE ALLOY	LEIAR25.R4-LEIT
LMMF	TRIMBLE ALLOY	TRM57971.00-NONE
LPGS	JAVAD TRE_3	JAVRINGANT_G5T-NONE
MAYG	TRIMBLE ALLOY	TRM59800.00-NONE
PIE1	JAVAD TRE_3 DELTA	ASH701945E_M-NONE
POTS	JAVAD TRE_3	JAVRINGANT_G5T-NONE
RGDG	TRIMBLE ALLOY	TRM59800.00-SCIS
SGOC	JAVAD TRE_3	JAVRINGANT_G5T-NONE
ULAB	JAVAD TRE_3	JAVRINGANT_G5T-NONE
UNB3	TRIMBLE ALLOY	TRM57971.00-NONE
URUM	JAVAD TRE_3	JAVRINGANT_G5T-NONE
WUH2	JAVAD TRE_3	JAVRINGANT_G5T-NONE
YAR2	TRIMBLE ALLOY	AOAD/M_T-NONE

## Data Availability

All data can be available from Multi-GNSS Experiment (MGEX) or by contacting the primary author.
